# Statistical models for identifying frequent hitters in high throughput screening

**DOI:** 10.1038/s41598-020-74139-0

**Published:** 2020-10-14

**Authors:** Samuel Goodwin, Golnaz Shahtahmassebi, Quentin S. Hanley

**Affiliations:** School of Science and Technology, Nottingham Trent University, Clifton Lane, Nottingham, NG11 8NS UK

**Keywords:** High-throughput screening, Cheminformatics

## Abstract

High throughput screening (HTS) interrogates compound libraries to find those that are “active” in an assay. To better understand compound behavior in HTS, we assessed an existing binomial survivor function (BSF) model of “frequent hitters” using 872 publicly available HTS data sets. We found large numbers of “infrequent hitters” using this model leading us to reject the BSF for identifying “frequent hitters.” As alternatives, we investigated generalized logistic, gamma, and negative binomial distributions as models for compound behavior. The gamma model reduced the proportion of both frequent and infrequent hitters relative to the BSF. Within this data set, conclusions about individual compound behavior were limited by the number of times individual compounds were tested (1–1613 times) and disproportionate testing of some compounds. Specifically, most tests (78%) were on a 309,847-compound subset (17.6% of compounds) each tested ≥ 300 times. We concluded that the disproportionate retesting of some compounds represents compound repurposing at scale rather than drug discovery. The approach to drug discovery represented by these 872 data sets characterizes the assays well by challenging them with many compounds while each compound is characterized poorly with a single assay. Aggregating the testing information from each compound across the multiple screens yielded a continuum with no clear boundary between normal and frequent hitting compounds.

## Introduction

High Throughput Screening (HTS) has been used for over a quarter century to identify new drug candidates^1^. During the same period, the scale of the compound libraries and the complexity of methods employed has increased^2^. Early in the development of HTS some compounds were noted to respond frequently^3^ leading to the concept of frequent hitters^3–6^ and pan assay interference compounds (PAINs)^7–9^. Although specific structural motifs are known to be problematic, they also are found in pharmaceutically useful compounds^10–12^. Practitioners of HTS have learned to recognize a wide range of chemical and physical processes leading to apparent activity in HTS assays^13^. The science of active compound detection and corresponding statistical practice developed early^14,15^ with many subsequent refinements^16–19^. The underpinning instrumental technologies of HTS have been influential for increasing the scale achievable in routine laboratory work and these technologies are widely deployed in the form of plate readers, lab robotics, and compound libraries accessible to researchers^20^. In addition, due to funding policies considerable HTS data is publicly available^21^.

Despite extensive development and some successes^1^, HTS remains subject to long-standing critiques related to productivity^22^. The slow pace of discovery has created an interest in strategies that repurpose existing drugs rather than seek new compounds^23^. Despite these limitations, considerable amounts of both public and private money are being invested in HTS and it is likely to remain a key aspect of drug discovery in the future.

The public investment and consequential data sharing provide a record containing many large primary HTS data sets. A previous investigation into a small number of these discovered an identifiable set of active compounds having excess variance many of which contained PAIN motifs^18^. This work generated an interest in understanding statistical behavior in HTS at scale. The goal was to fill a gap in the literature documenting the distributions observed in HTS, assess conformity with assumptions/expectations, and understand the characteristics of active, inactive, and inconclusive compounds. Here we investigated sampling of chemical space, screen and compound level metrics, and models of frequent hitters. While some of the results noted here may be known in industries involved in screening directly, we have not seen similar results presented previously.

## Theory and definitions

This section considers term definitions, screen models, compound models, and rank order models. In the screen model section, we consider the binomial survivor approach to identifying frequent hitters, adaptation of that model to consider multiple probabilities, and additional statistical functions used to model screen level probabilities in the data analyzed. In the compound model part, we present an empirical gamma distributed model of active assignments and approaches to distributing chemical attributes to model observed distribution behavior. Background on rank order models is provided to assist understanding this presentation.

### Definitions

Compounds were considered ***active***, ***inconclusive***, or ***inactive*** based on assignments in the data sets. ***Active*** designations are used interchangeably with ***hits***. Compounds were deemed ***frequent hitters*** if they hit more often than expected based on a model of active behavior. When speaking of individual compounds, we restricted discussion to compounds tested 50 or more times. This threshold is arbitrary. Hit rates vary in assays with 0.5–2% of compounds found active^24^. True positives are less common with few or none of a set of hits genuinely interacting with a target^25,26^. A compound generating a true hit once in fifty screens (2%) will be poorly characterized using 50 as a cutoff. An ***infrequent hitter*** is one found to be active fewer times than expected and a ***normal hitter*** is a compound meeting statistical expectation.

### Screen models

The binomial survivor function (BSF) was proposed to identify frequently hitting compounds using information from multiple screens^6,27^. This model considers each test of a compound as a trial with some probability, *p*, of success. The value of *p* is estimated from hit rates in multiple screens. Across *n* such trials the probability a compound is active *k* times can be computed using the binomial probability mass function,

1 $$ BSF\left( {k,n,p} \right) = \left( {\begin{array}{*{20}c} n \\ k \\ \end{array} } \right)p^{k} \left( {1 - p} \right)^{n - k} . $$

Compounds with values of pBSF ≥ 2 (99% confidence) were considered diagnostic of a compound being a frequent hitter compound where pBSF = − log_10_(BSF)^6,27^. The BSF model assumes *p* is single valued and only considers compounds being active more times than expected (frequent hitters). Compounds can also be assigned active too few times making them infrequent hitters.

Multiple probabilities can be considered by adapting the BSF model. When *N* screens are performed each having a probability, *p*_i_, this can be simulated by *N* sets of trials with the number of trials equal to the number of compounds in each screen. The values of *p*_i_ can be estimated from the fraction of active compounds in each screen. This type of process is described by the Poisson-binomial distribution^28–30^.

2 $$ PB\left( k \right) = \mathop \sum \limits_{{A \in F_{k} }} \mathop \prod \limits_{i \in A} p_{i} \mathop \prod \limits_{{j \in A^{c} }} \left( {1 - p_{j} } \right) $$

$F_{k}$ represents every subset of *k* integers contained in {1 …. *n*} where n is the number of trials and *k* the number of successes.

Such a framework allows observed results to be simulated when the probability is not constant. There is additional complexity in the data considered here due to the number of tests on each compound varying in a complex way (see Fig. 2). For this reason, each compound was simulated as the number of successes, *s*, in *j* tests done on each of the *n* compounds in *i* screens. The probability of success for each test was drawn from a function describing the distribution of *p*_i_.

The distribution of *p*_i_ values obtained from the percentage of active compounds in each screen was modelled by a generalized logistic distribution^31–33^ (LD) after logit transformation.

3 $$ LD\left( {p; \theta , \sigma , \alpha } \right) = \frac{\alpha }{\sigma }\frac{{e^{{ - \frac{p - \theta }{\sigma }}} }}{{\left\{ {1 + e^{{ - \frac{p - \theta }{\sigma }}} } \right\}^{\alpha + 1} }} $$

where *θ*, *σ*, and *α* are the location, scale and shape parameters.

### Compound models

Another statistical tool to model active assignments in screens is based on the Gamma distribution (GD) with the following form

4 $$ GD\left( {x;\alpha ,\beta } \right) = \frac{{\beta^{\alpha } x^{\alpha - 1} e^{ - \beta x} }}{{\left( {\Gamma (\alpha )} \right)}}, $$

where *α* and *β* are parameters of the Gamma distribution and can be parameterized such that *α* = mean^2^/variance and *β* = mean/variance. Once the parameters are found, gamma distributed random numbers can be generated to simulate data and the resulting simulation compared to the binomial models and to the observed data.

Compounds have a range of attributes that combine to generate responses in screens and these attributes can be modelled using statistical mechanisms. These attributes might include approximate limits on drug-like chemical space such as Lipinski’s rules of 5^34,35^. Other features might be useful building blocks^12^, privileged scaffolds^4^, PAIN motifs^9,37^, and other related chemical attributes. A bottom-up model can distribute attributes by assuming initially that a randomly selected compound has a probability, *p*_a_, of having a detectable attribute based on a binomial trial. If it has one attribute, then it has the same probability of a second, and so forth. This is like flipping coins weighted by *p*_a_ and accumulating attributes while a continuous run of successes is obtained. Assuming all attributes have a single probability is clearly an over-simplification but provides a starting framework for a parsimonious model of compound behavior. The number of successes, *k*, after *r* failures, in such a process is modeled by the negative binomial (NB) distribution.

5 $$ NB\left( {k,r,p_{a} } \right) = \left( {\begin{array}{*{20}c} {k + r - n} \\ k \\ \end{array} } \right)p_{a}^{k} \left( {1 - p_{a} } \right)^{r} $$

To model the number of assayable attributes in a set of compounds we parameterized the NB to only allow a single failure, *r* = 1. Once the attributes are distributed, each attribute provides a chance of being designated as active in any screen based on the probability of success. This second stage considers the attributes accumulated according to Eq. (5) to be like tickets for a lottery (a screen) with a probability of success for each ticket. This model can then be compared to a rank order presentation of the number of hits a set of compounds accumulate.

### Rank order models

Because the literature of drug discovery has been interested in PAINs^8,9,38–40^, frequent hitters^3,6^, and related effects^23^ it is convenient to look at data in rank order^41–48^ to focus on compounds appearing multiple times across screens and the variability in the hit percentage across the data sets. A rank order model sorts the data by a parameter, for example the number of tests on each compound, and seeks to model the shape. A three-parameter model for such data has been proposed as an extension to Zipf’s law^49^.

6 $$ y\left( r \right) = A\frac{{\left( {N - r + 1} \right)^{b} }}{{r^{a} }} $$

In this expression, *A* is a scale constant, *a* determines the curvature at low values of *r*, and *b* describes the curvature at large values of *r*. Specific values of *a* and *b* are related to specific distributions. For example, when *a* = *b* = 0 the uniform distribution is obtained^47^.

## Results and discussion

### Overview of the 872 screens

The 872 HTS data sets examined all contained tests on 50,000 or more compounds. The data include assay results from 1,759,553 compound identification (CID) numbers. These represented approximately 1.7% of the ~ 103 million compounds on PUBCHEM and about 1.2% of the ~ 145 million CID numbers assigned as of 24/3/2020. The percentage of active compounds ranged from 0% (AIDs 592, 901, 1019, 1027, 1722, 1766) to 69.62% (AID 1996, solubility assay; 40,282 active out of 57,859 compounds tested). The six with no actives included spectroscopic profiling tests (AID 592) and assays against disease targets (AID 901, inositol monophosphatase). These 872 screens contain 223,573,071 compound assays against valid CID numbers which represented approximately 83% of the bioassays on PUBCHEM as of 24/3/2020. There were 1,559,098 assays resulting in active designations (0.782% of total) and 2,560,020 inconclusive tests (1.28% of total). These counts do not include a small number of compounds for which no CID number was included. The active designations represent 374,431 different compounds (21.2% of all compounds tested) and there were 431,189 inconclusive compounds (24.5% of compounds).

### Active compounds in screens

**Figure 1:**
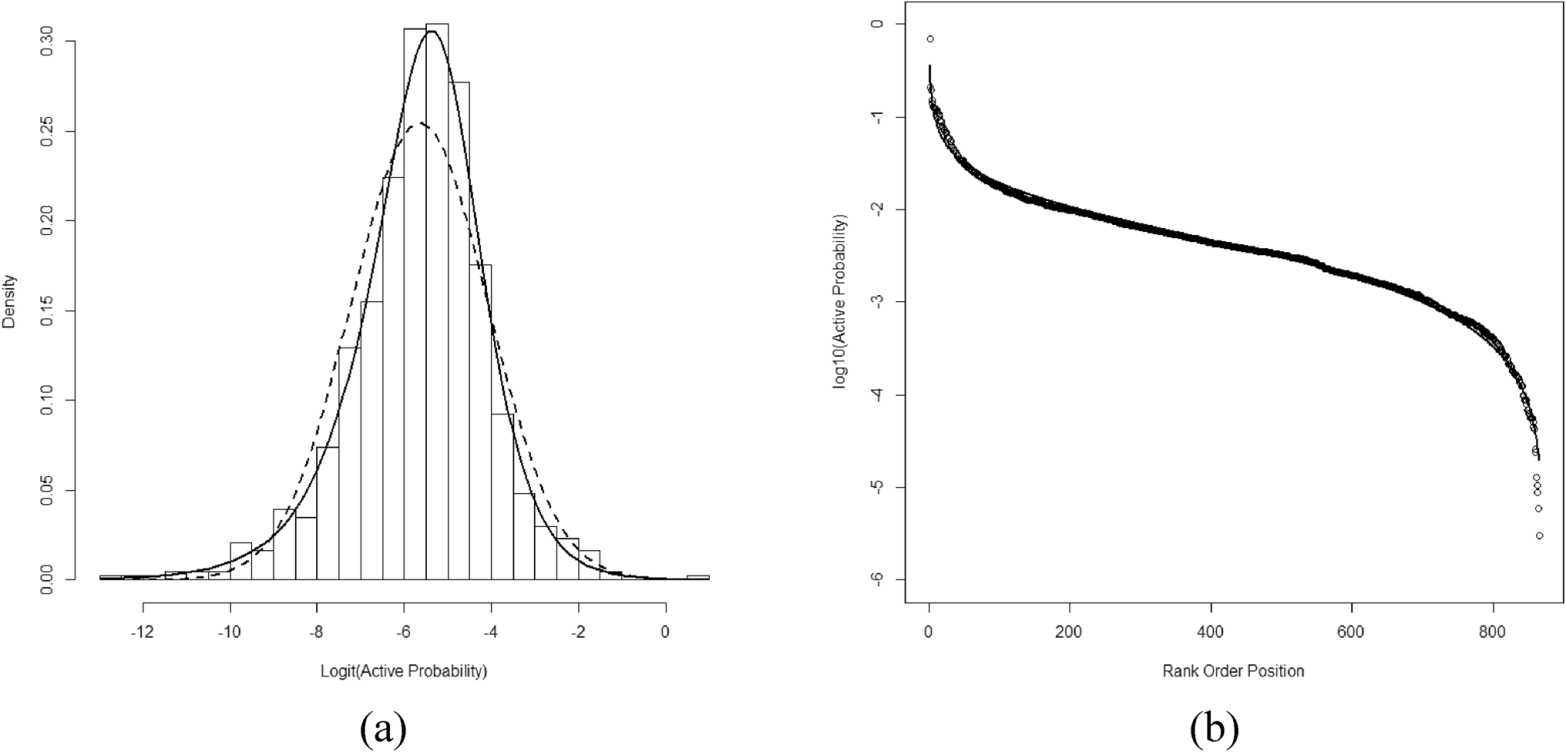
The density histogram (**a**) of logit transformed probabilities when compared to a generalized logistic distribution (solid line) and a normal distribution (dashed line). The rank sorted (**b**) probabilities from the 872 screens fit well to the three-parameter model Eq. (5) (solid line).

The fraction of active compounds in screens has been considered a probability when trying to identify frequent hitters^6,27^. The wide range in the observed percentage of active compounds seen here suggested that no single probability defined the behavior over the series of screens. Although the average probability was 0.0078, this number had considerable variance. To better understand the distribution of the expected number of actives, we investigated the normal, skew normal, beta, logistic, and generalized logistic distributions as candidate distributions describing these probabilities. The logit transformed probabilities gave good correspondence with a generalized logistic distribution^33^ (GLD) with location = -5.0587, scale = 0.6958, and shape = 0.6333 (Fig. 1a) in comparison to a normal distribution. The rank ordered data fit well to Eq. (5) (Fig. 1b) with parameters: *a* = 0.6084 ± 0.0068, *b* = 1.183 ± 0.0074, and *A* = 0.000121 ± 0.000091. Simulations using GLD random numbers followed by inverse logit transform were nearly identical to the data with reasonable recovery of rank order parameters (*a* = 0.64 and *b* = 1.01). These *a* and *b* values occupy a region that is currently not well documented^50^. Descriptively, *a* and *b* are related to the skewness and shape of the distribution with the *a* and *b* parameters related to the left and right sides of the distribution, respectively.

The distribution of active probabilities (Fig. 1a) provides the information needed for a generalization of the binomial survivor model based on the Poisson-binomial distribution to build prospective models of large HTS programs consisting of hundreds of campaigns. The shape of this distribution is critical for appropriate cost–benefit analysis of publicly funded HTS as it enables estimations of the scale in terms of campaigns required to find some number of lead compounds for an arbitrary set of diseases. In combination with a more holistic view of the entire drug discovery process, it could allow reasonable predictions of the resources needed from beginning to end of the process. To the extent these 872 screens are representative of public HTS campaigns, it is the best data available for modeling future campaigns.

### Frequency analysis of tested compounds

**Figure 2:**
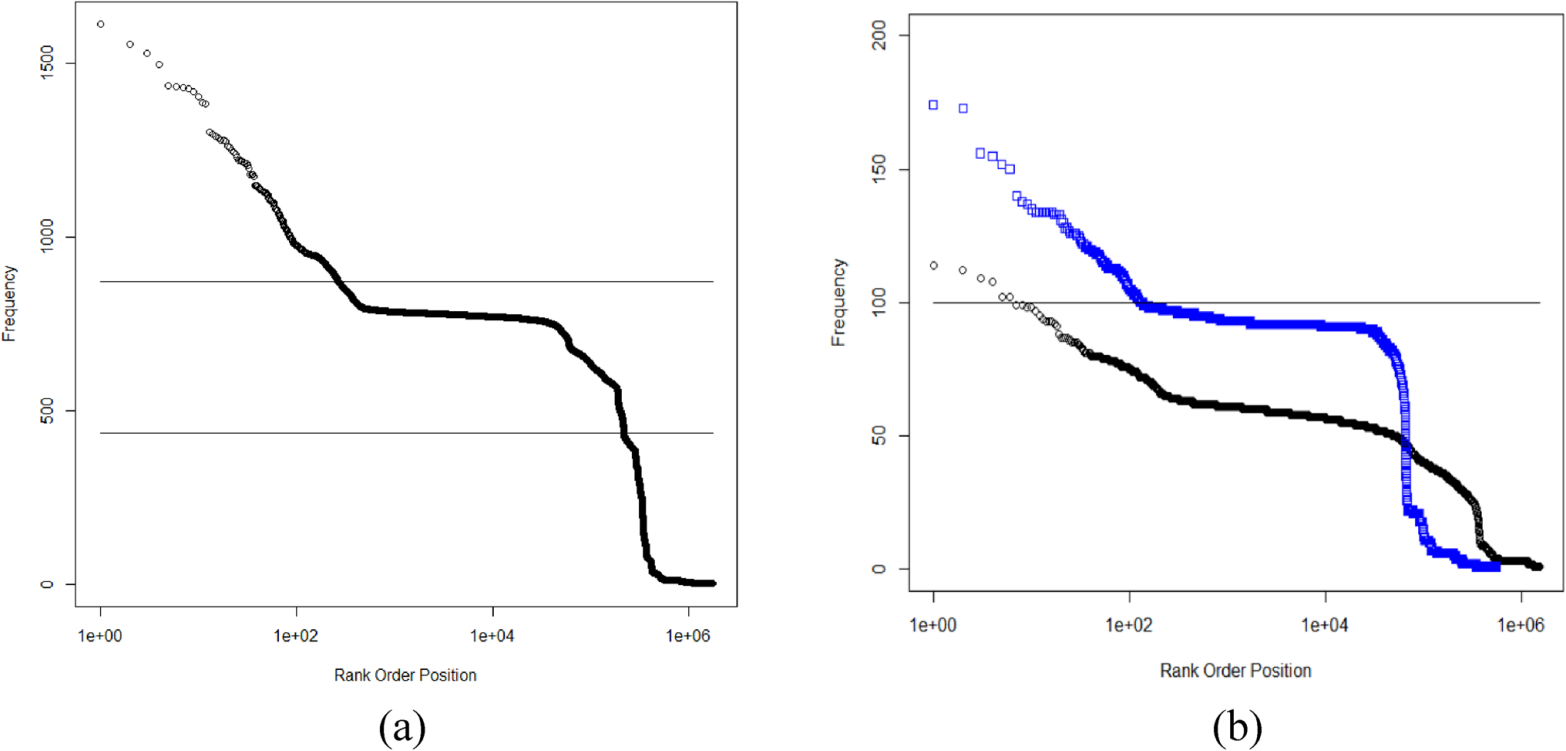
Rank order distributions of the number of times each compound was assayed. Panel (**a**) represents all 872 screens and shows the key features of the compounds tested. Panel (**b**) is a comparison of the first (blue squares) and last (black circles) 100 screens (**b**). Values above the lines at 872 (**a**) and 100 (**b**) represent compounds tested more times than there were screens. Values above the line at 436 in panel (**a**) represent compounds having greater than 50% likelihood of being tested in any screen.

The frequency with which each compound was tested was noted by the PUBCHEM CID number (Fig. 2). The frequency with which compounds were found to be inactive had a similar shape and structure (not shown). The rank ordered distribution shows a prominent feature due to a subset of the compounds appearing repeatedly within single data sets (Fig. 2). A total of 275 compounds were tested more than 872 times. The most tested compounds were a known approved drug: the anti-depressant maprotiline hydrochloride (CID 71,478; 1613 tests) and the serotonin reuptake inhibitor fluvoxamine maleate (CID 9,560,989; 1555 tests). Maprotiline hydrochloride was also inactive the greatest number of times (1544). Several of the most tested compounds are approved drugs.

Investigation of the multiple repeats of specific compounds revealed flaws in the construction of compound libraries as well as problems with reporting stereochemistry to PUBCHEM. Two examples are worth considering: 4-aminobutanoic acid (GABA; CID119) and inositol (CID892). GABA appears multiple times in compound libraries and was tested 893 times in the 872 data sets. It appears four times in the AID175 screen under four different SID (substance identification) numbers which are all mapped to a single CID. This replication remained at the end of the period. GABA appeared 63 times in the first 100 screens and 66 times in the last 100. For GABA and similar compounds, better library management could reduce the cost of repeatedly assaying such compounds in single screens.

More problematic is the case of inositol. Inositol appears nine times in the two screens represented by AID 175 and AID 248 and 1067 times in the 872 screens. Inositol has nine stereoisomers and some of these are clearly designated in the names provided with the SIDs to PUBCHEM. PUBCHEM had eight CID numbers associated with inositol, however, six were deuterated or tritiated isotopologues. In AID 175 and AID 248 there were eight different SID numbers submitted with structures all mapping to CID 892. The names provided with the eight SIDs sometimes refer to specific forms leaving ambiguity between name and structure. The 1067 repeats assigned to CID892 may reflect this ambiguity and the tests were made on specific inositols but the stereochemical information was subsequently lost during reporting to PUBCHEM. All 9 inositols currently appear as synonyms for CID 892 along with hundreds of other names. Given the importance of stereospecific effects^51^, this loss is concerning and researchers trying to mine the PUBCHEM data base may be unaware of this problem. Submitters need to be more careful about the structural information they provide to better deal with stereochemistry and greater care would enhance the value of the PUBCHEM data base.

The compound test frequency rank order distribution (Fig. 2a) includes two other notable groups of compounds. One group of compounds is made up of the same core libraries tested over and over and another group tested 3 or fewer times. As an example of the former, there were 218,998 compounds tested over 436 times. This group represents 62% (139,313,672) of the 223,573,071 tests. The bulk of tests appear to represent a small core of compounds tested repeatedly. A similar portion (235,292 compounds, 13.4% of the total) were tested only once (0.1% of tests). It is unlikely this represents the best approach to compound library design and management. More equitable testing and expansive sampling of chemical space might help find more new drugs.

This structure changed somewhat over the 15 years represented in the 872 data sets (Fig. 2b). The change we ascribe to an increased number of screening labs with more variation in libraries, however, each lab is retesting its core library repeatedly. The scale of the number of compounds tested increased roughly a factor of 3 over the period with increased diversity during the last 100 screens. Based on this evidence, we concluded that this series of HTS experiments taken in aggregate includes extensive compound repurposing at scale^23^. It also includes direct drug repurposing (e.g. the most tested compound maprotiline). In the 872 HTS data sets, core libraries are repeatedly tested against new targets. When these are looked at in isolation, it may seem reasonable to redeploy an existing library against a new target, but in aggregate the likelihood that discoveries will be made of fundamentally new classes of drugs in the core of repeatedly tested libraries is small. At the same time, more attention needs to be paid to the compounds tested a small number of times. How these are introduced and managed is particularly important as it is arguably the part of these screens representing drug discovery rather than compound repurposing at scale (Table 1).

**Table 1:** Date markers for when CID numbers were created in the PUBCHEM database.

CID number	Date added
1	Jun 23, 2005
10,000,000	Oct 25, 2006
20,000,000	Dec 5, 2007
40,000,000	May 30, 2009
60,000,000	Aug 20, 2012
80,000,000	Oct 19, 2014
100,000,000	Dec 11, 2015
120,000,000	May 15, 2016
130,000,000	Oct 7, 2017
140,000,000	Dec 6. 2019

**Figure 3:**
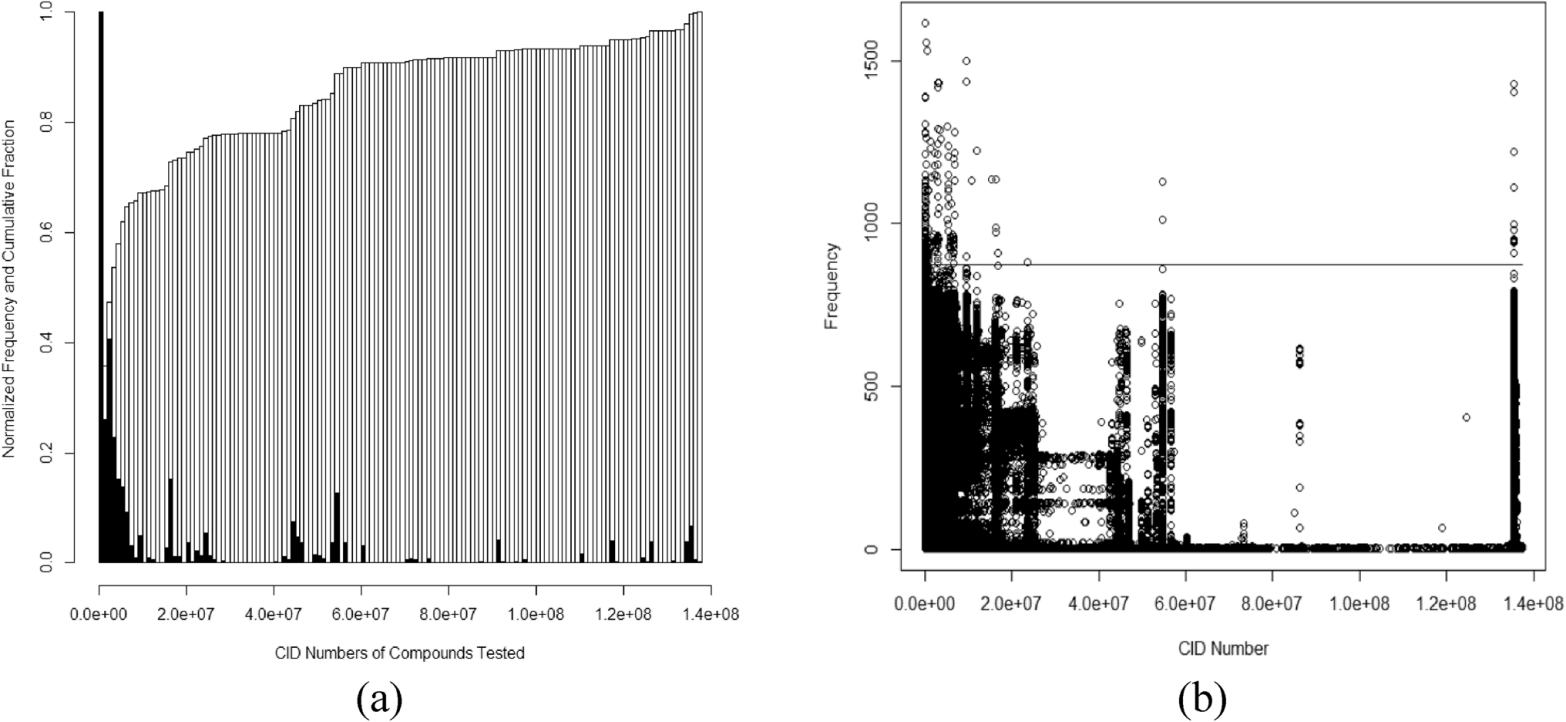
Maximum normalized histogram and cumulative sum of CID numbers (**a**) and plot of test frequency against CID number (**b**). Compounds above the line in panel (**b**) were tested more than 872 times. Note the CID number date markers in Table 1.

A further issue became apparent when assessing how CID numbers were distributed (Fig. 3). CID numbers are assigned sequentially which provides an approximate timeline for when the compounds were added to the PUBCHEM data base (Table 2). There are some limitations to this approach because some CID numbers added recently represent compounds that were used in early screens. One such example is CID 135,400,595 which was added in 2019 but used as early as AID368 (deposited in 2006). Despite this limitation, constructing a plot of the frequency of tests against CID number (Fig. 3) gives insight into the extent to which chemical space (as represented by CID numbers) is being sampled (Fig. 3a) and tested (Fig. 3b). It is neither sampled nor tested uniformly with large sections almost untested. Over 58% of compounds tested were drawn from the first 10 million compounds added. The aggregate library of 1,759,553 compounds tested in these screens is clustered into blocks as are the number of times tested. In the absence of evidence to the contrary, there is no reason to believe that the poorly sampled sections of chemical space are less likely to contain pharmacologically useful compounds. There are also very specific CID number ranges that contain nearly all excessively tested compounds (> 872 tests).

### Frequency analysis of active and inconclusive compounds

**Figure 4:**
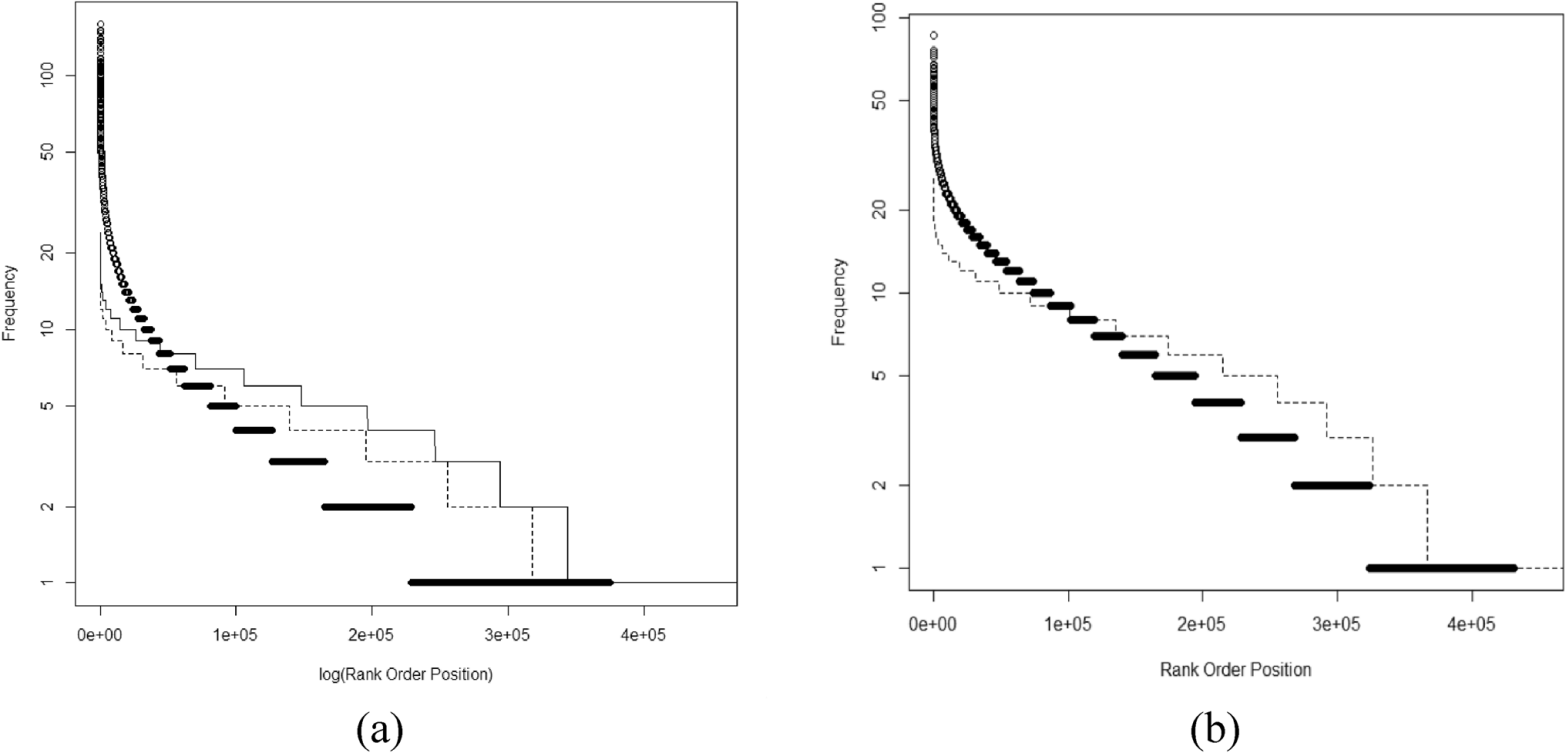
Active (**a**) and inconclusive (**b**) rank order distributions obtained from the 872 screens. The Poisson-binomial (solid line) and binomial (dashed line) models cross the observed active distribution (black circles) indicating the existence of a group of infrequent hitters (note the log scale). The binomial simulations used *p* = 0.0078 (active) and *p* = 0.0128 (inconclusive).

Application of the binomial survivor function^6,27^ such that a frequent hitter was any compound with pBSF > 2 found 42,264 compounds (11.3% of actives) meeting these criteria with more than 50 tests. We investigated further using two binomial simulations (Fig. 4a). The first model consisted of a binomial with a single probability (0.0078). The second was a Poisson-binomial model using probabilities drawn from the generalized logistic distribution. These models were used to simulate the structure of the active rank order distribution. When compared to the observed active distribution (Fig. 4a), both simulations indicated a large group of compounds hitting above expectation. The Poisson-binomial generated fewer frequent hitters but the improvement was marginal. Both models gave a poor representation of the observed data. Both models generated a much larger group of infrequent hitters. Infrequent hitters have not been described previously and they are important for understanding HTS. Specifically, binomial models based on screen level probabilities (Fig. 1) do not predict how hits distribute among the compounds in a screen. These probabilities are not equal due to the chemical properties of the compounds tested. The literature contains extensive discussion of PAINs and frequent hitters, but the chemistry of infrequent hitters also needs investigation.

Similar behavior was seen in the distribution of inconclusive compounds (Fig. 4b). The scale of this group was unexpected and to our knowledge there has been little discussion of this group of compounds. A single valued binomial simulation gave a poor representation of these compounds with many “frequent inconclusive” and “infrequent inconclusive” compounds. Inconclusive compounds represent a slice of the response distribution between the threshold for being “active” and the remainder of the distribution. As such, true hitters are expected to be concentrated above the active threshold and less prevalent in the inconclusive section of the distribution and therefore fit a binomial survivor model better. There was little evidence supporting this (Fig. 4) and the binomial simulation of the inconclusive compounds was unsatisfactory. It is unclear whether inconclusive compounds are investigated seriously during lead generation but from a model building point of view they are essential. Inconclusive designations represent a different section of the distribution of measured values from the active tail and need to be included in a self-consistent model of HTS.

### Models of active designations

The failure of the binomial models based on *screen* probabilities to provide a model of active compound frequencies required alternatives based on compound behavior. Two types of models were investigated to simulate the frequency of active designations across the 872 HTS data sets. Type I sought to build a model of compound activity giving chemical insight into what makes a compound hit. The goal was to simulate the results for randomly selected molecules drawn from a chemical space. This approach assumes that the compounds in the study are representative. Successful model construction could help decide whether HTS should grow ever larger in scale or should be smaller with more randomness in compound selection. Type II models sought improved statistical description reducing the proportion of infrequent hits.

**Figure 5:**
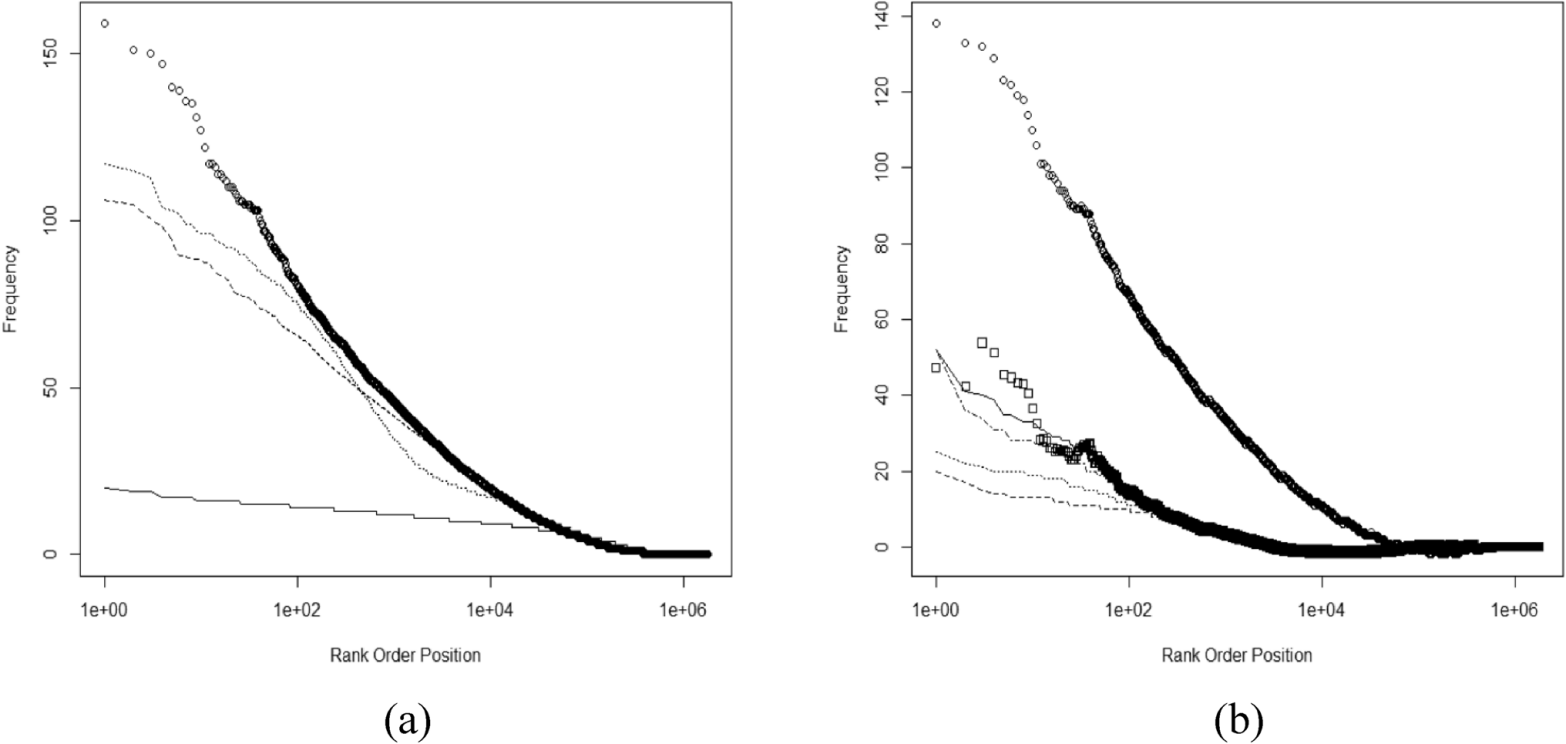
Rank order presentation of active designations in the 872 screens (**a**) compared to the BSF (solid line), gamma (dashed line), and attribute (dotted line) models. The residuals (data–model) (**b**) generated from the binomial survivor (circles) model resulted in a greater number of both frequent hitters and infrequent hitters than the gamma model (squares). The residuals from the gamma model could be approximated by summing over a set of negative binomial attribute distribution processes. This is illustrated for one (dashed line) to four (solid) attributes.

An initial type I model was built on the following assumptions: (1) compounds have attributes that can be detected in a screen; (2) attributes are accumulated following a negative binomial process with *r* = 1 and *p*_1_ = 0.50 with *p*_1_ found by least squares minimization; (3) each attribute may be detected (result in active designation) with a binomial probability, *p*_2_ = 0.01. A small group would be expected to get a string of 18–20 successes (attributes) in a row and 50% would be expected to fail on the first attempt. The attributes distributed in this manner might include Lipinski’s five^34^, privileged scaffolds^4^, or PAIN motifs^9,37^. For example, if all compounds on PUBCHEM were sampled, there is some probability a randomly sampled compound has a molecular weight under 500. This model is simple but provides a framework for a probabilistic approach to estimating compound behavior in screens. This model (Fig. 5a, dotted line) gave a better approximation to the observed behavior of the compounds in rank order than the BSF model.

We then investigated descriptive models for this data. A gamma distributed simulation (Fig. 5a, dashed line) parameterized by shape (mean^2^/variance = 0.0755316) and rate (mean/variance = 0.085242) worked for much of the range leaving a much smaller (~ 1000) frequent hitter group (Fig. 5b) and fewer infrequent hitters than the BSF model. The residual sum of squares for the binomial and attribute models were 27 and 3.3 times greater than the gamma model, respectively. The residuals from the gamma model could be approximated by summing attributes. For example, one to four attributes gave the results shown in Fig. 5b. While not definitive, the analysis made clear that considering the behavior of compounds rather than screens is required to understand active compounds. In the context of HTS, a clear interpretation of the gamma distribution is difficult, however, it was clearly better than any of the binomial models tested. The key limitation of the binomial models using screen derived probabilities is the assumption that all compounds have equal opportunities in a screen unless they are part of the select group of frequent hitters. This assumption cannot be supported by the data.

### Compound hit probabilities

**Figure 6:**
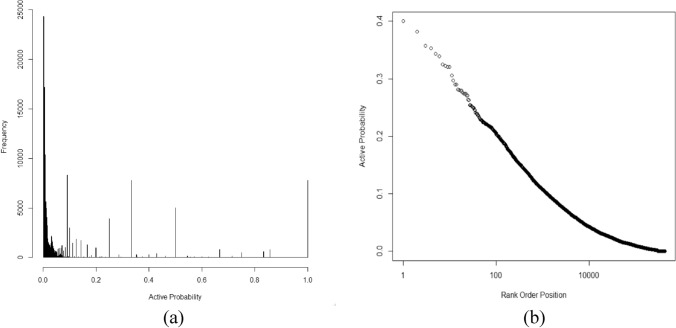
The histogram of *p*_*d*_ values (**a**) restricted to 0 < *p*_*d*_ to highlight the structure of the histogram and rank order presentation (**b**) of *p*_*d*_ for all compounds tested more than 50 times.

Compound behavior can be considered in terms of the total number of hits (Fig. 4) or by the fraction of screens where a compound was active. The former is a better indicator of total follow on cost and the latter a better indication of compound behavior. Individual compound probabilities can be investigated to see if a clear divide exists between normal and frequent hitters. The histogram of compound hit probabilities for *p*_*d*_ > 0.0 (Fig. 6a) gives a spiky appearance with peaks in the histogram at 1, 0.5, 0.33, 0.25. 0.2 etc. corresponding to 1 active test in 1, 2, 3, 4, 5, … measurements. To avoid this and similar issues, the rank order presentation (Fig. 6b) was restricted to compounds tested 50 or more times (420,572 compounds). The histogram (Fig. 6a) was neither normally distributed around some constant nor obviously bimodal and there was a nearly continuous range of *p*_*d*_s (Fig. 6b) giving no clear boundary between normal and frequent hitting compounds.

Figures 1 and 6 reveal a limitation in the current approach to HTS. A single HTS screen is an extremely poor way to characterize compounds, particularly if each is tested once. It is an extremely good way to characterize an assay response. A compound tested once is poorly understood. However, an assay challenged with 100,000 compounds has been thoroughly tested.

### Characteristics of frequent hitters

The chemical characteristics of frequent hitters were assessed based on the number of times they were deemed active relative to the gamma model in Fig. 5a. We chose hit numbers, rather than hit probabilities since each hit would have required further workup and cost. For example, the most frequently active compound, 3-Methyltoxoflavin (CID 460,747, 159 times), has been patented as a treatment for tuberculosis but may have been considered multiple times for other conditions. The second most active compound (toxoflavin; CID 66,541; 151 times) features in multiple patent applications. Forty-four compounds were active over 100 times. Similarly, the first 100 were active over 80 times each and the 1000 most frequent hitters were all active 45 or more times. For scale, 374,431 different compounds were deemed to be active 1,559,098 times. Follow on costs associated with these active designations was probably high.

**Figure 7:**
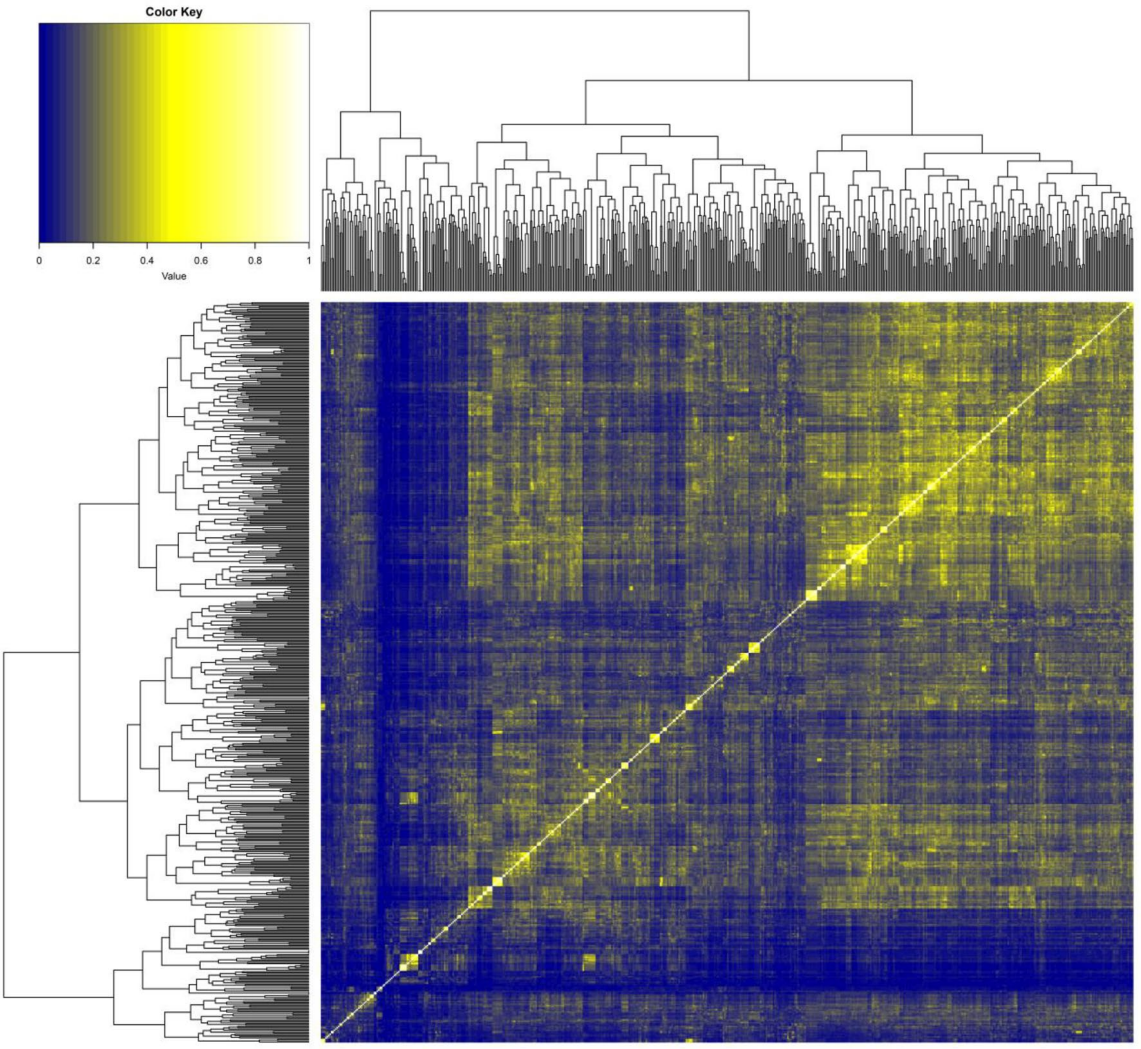
Heat map of Tanimoto similarity of the 568 compounds within the 1000 most active compounds not matching PAIN filters or considered inorganic.

Investigation of the 1000 most active compounds yielded 429 suspected PAINs, 568 not matching any PAIN^37^ motif, and 4 inorganic or otherwise unclassified compounds (e.g. CID 44,202,984). Of the 568 compounds not matching current PAIN motifs, 80 contained other undesirable substructures. These 568 are of interest because they represent compounds that could be privileged structures^3,4^, PAINs^7–9^, or both. To assess this, the 541 without any suspected PAIN motifs were examined for structural similarity and presented as a heatmap (Fig. 7). Each pixel in the heatmap represents the Tanimoto similarity between two compounds with brighter pixels representing greater similarity. The heatmap shows many recognizable clusters as square along the diagonal with three examples presented in Table 2. All 568 compounds deserve further scrutiny and the structural motifs defining the clusters identified. A full annotated list the 1000 CID numbers appears as supplementary material S1.

**Table 2:**
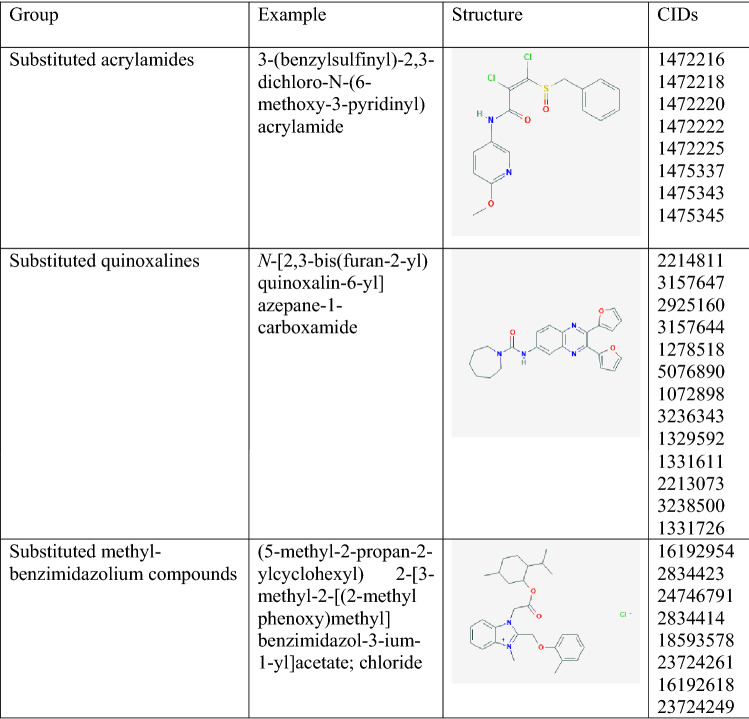
Example Motifs found in clusters.

Each compound was active over 45 times.

Across the 872 screens, compounds were repeatedly tested but subsequent analysis does not appear to extend much beyond single screens. This leads to multiple retesting of frequent hitters, PAINs, potential PAINs, and otherwise promiscuous compounds as well as missing an opportunity for discovering new scaffolds. Although some of these multiply active compounds may have useful characteristics, in the absence of a more coherent approach, repeated retesting is not going to discover this. The scale of the problem needs to be understood. Within this data set there were 37,050 compounds deemed active 10 or more times. There is no chemical overview of either the modes of action of these compounds or their specificity (if any). It is unclear whether they should be purged from libraries or built into smaller scale libraries as a pre-HTS compound repurposing stage.

## Conclusions

Most of the HTS data represented here is compound repurposing at scale (Fig. 2) and assay characterization. Many compounds were repeatedly retested and subsequently treated screen by screen. The multi-screen information is the most valuable for chemists. It is unclear whether industrial screening companies provide this information to their clients. It is also unclear whether large scale international screening programs are being set up so compound behavior can be transparently assessed.

Repeated retesting raises issues similar to the multiple comparisons problem; with enough tests eventually all the compounds will be designated as active. Across the 1.7 million compounds here, there is a large amount of prior information of varying quality but little evidence it is used effectively. For example, a compound with 100 active designations may be understood sufficiently for decisions to be made about its behavior. It is not clear why such compounds remain in HTS libraries. Compounds tested only a few times are not well characterized and it is not clear why so many compounds do not remain longer. Information about compounds from previous screens needs to be used more effectively.

Future studies trying to discover new drug compounds should consider sampling chemical space in a more strategic way (Fig. 3). A 1.7 million sub-sample of 100 million items could be an excellent sample if done carefully. However, to the extent that PUBCHEM represents a view of a chemical space, most compounds tested are in the first 10 million added and much of the remainder is in specific regions. These same restricted regions are most likely to be repeatedly or excessively tested. It is worth looking more closely at library construction. Is the best predictor of compound inclusion today whether it or a similar compound was there 15 years ago? If these isolated portions of PUBCHEM chemical space really contain the best hope for new drugs, then they should be used. However, it is unclear this hypothesis has been rigorously tested. For the remaining less tested compounds, the process by which they are included, tested a few times, and discarded is unclear. For some, a single test was considered enough. For others, no decision was made even after 1613 tests.

The binomial survivor model is an extremely useful and falsifiable model. As clearly as it identifies frequent hitters, it also generates a much larger pool of infrequent hitters (Fig. 4). Considering a distribution of probabilities representing these screens rather than a single probability does not mitigate this problem. The chemical behavior of infrequent hitters deserves as much attention as frequent hitters. A self-consistent chemical model of what makes a compound hit is essential to understanding how attributes contributing to activity are distributed in chemical space. Future models should be able to identify infrequent hitter chemical motifs which may also be the keys to making compounds less toxic.

Aspects of the rank order active designations (Fig. 5) can be generated by considering attributes randomly distributed in a collection of compounds by a negative binomial process. This attribute model also generates excessive infrequent hitters. A gamma distribution parameterized by shape and rate was a better predictor of the number of active designations. Both models performed better than the BSF model indicating that compounds do not have a binomial probability of being found active in a screen. With enough tests the probability that a compound will be active can be quantified (Fig. 6), but when investigated, a continuum between normal and frequent hitting compounds was found. There was no clear boundary between them.

Nearly half of the 1000 most active compounds in the 872 screens were suspect PAINs. The remainder contained clusters of compounds which should be examined for new motifs either to decorate (privileged structures) or avoid (PAINs). In future studies, it will be useful to investigate the strength of the responses to these compounds and if they respond preferentially in particular types of assays. The presence of these compounds indicates that insufficient effort is being made to understand the behavior of compounds across multiple screens.

This study assumed that the process of assigning active compounds was done perfectly. This needs to be revisited in future work. To better understand chemistry rather than assays, future studies should be looking to challenge compounds with multiple assays, rather than the other way around. While there is considerable public data providing the information for some compounds, this set of 872 HTS studies were not designed to characterize chemical behavior and are doing it poorly.

## Methods

### Data sets

Data were obtained from PUBCHEM and included all Assay Identification (AID) numbers having more than 50,000 compounds in the data base on 19 March 2020. This yielded 872 usable data sets. A few had to be discarded because they did not include decisions on activity. The data cover a period of approximately 15 years with the first screen in the study (AID155) deposited August 15, 2004 and the last (AID 1,347,076) deposited on July 31, 2019. The data were downloaded 19 March 2020.

### Statistical analysis

The data were downloaded and analyzed using R^52^ (Version 3.6.2) running within R-studio (version 1.2.1335) with packages dplyr^53^, car^54^, brms^55^, moments^56^, gplots^57^, ggplot2^58^, ChemMineR^59,60^, and glogis^32^. The active, inconclusive, inactive, and all CIDs were identified and collated across the 872 data sets by CID number. Tests for PAINs were done using the FAF-Drugs4^37^ portal. Simulations were done using either random number generators (to simulate individual compounds in screens) or using numerical computations of the distributions. These were done using the R-packages noted and all the code needed to reproduce the study has been provided.

## Supplementary information

Supplementary file1

Supplementary file2

Supplementary file3

Supplementary file4

Supplementary file5

Supplementary file6

Supplementary file7

Supplementary file8

Supplementary file9

Supplementary file10

Supplementary file11

Supplementary file12

Supplementary file13

Supplementary file14

Supplementary file15

Supplementary file16

## Data Availability

All the data analysed here is freely available via PubChem. An annotated listing of data and code files has been provided (TitlePage_SI.pdf) along with the R-scripts (1_download_rev1.R, 2_Siftdownload_rev1.R, 3_CombineTables_rev1.R, BinomialSurvivor_rev1.R, Fig. 1_rev1.R, Fig. 2_rev1.R, Fig. 2b_rev1.R, Fig. 3_rev1.R, Fig. 4_rev1.R, Fig. 5_rev2.R, Fig. 6_rev1.R, and Fig. 7_rev1.R), specification file (AID_NUMBERS_rev1.csv), an annotated list of the most frequently active compounds (Top1000Actives_all872.csv) and R readable data tables.
