# Supplementary material for: Statistical models for identifying frequent hitters in high throughput screening
Source: Sci Rep. 2020 Oct 14;10:17200. doi: 10.1038/s41598-020-74139-0 (PMC7560657; doi:10.1038/s41598-020-74139-0)
Supplement: Supplementary file 15 — Supplementary file15 [file 41598_2020_74139_MOESM15_ESM.docx]

Supplementary Information for Statistical Models for Identifying Frequent Hitters in High Throughput Screening

Samuel Goodwin, Golnaz Shahtahmassebi, Quentin S. Hanley ^*^

*School of Science and Technology*

*Nottingham Trent University*

*Clifton Lane*

*Nottingham NG11 8NS*

*United Kingdom*

[quentin.hanley@ntu.ac.uk](mailto:quentin.hanley@ntu.ac.uk)

Telephone: +44 (0) 115 848 3536

*** Corresponding author:** Q. S. Hanley

**Annotated Listing of Additional Files**

The R-script, **1_download_rev1.R**, will download the files from PUBCHEM and save them into the default location of an RStudio install. It requires the specification file, **AID_NUMBERS_rev1.csv**, which specifies the data sets by AID number and provides some additional data.

The R-script, **2_Siftdownload_rev1.R**, tabulates the number of tests, the number of active designations, the number of inconclusive designations, and the number of inactive designations in the downloaded data files by CID number and saves them to tables. This file can be modified to analyse specific ranges (e.g. first and last screens in the specification file).

The R-script, **3_CombineTables_rev1.R**, combines the tables into a single table. The data table **CombTab_872.tab** was created from this script.

The R-script, **BinomialSurvivor_rev1.R**, computes pBSF values for the compounds in **CombTab_872.tab**.

The remaining R-scripts (**Figure1_rev1.R**, **Figure2_rev1.R**, **Figure3_rev1.R**, **Figure4_rev1.R**, **Figure5_rev2.R**, **Figure6_rev1.R**, and **Figure7_rev1.R**) perform the analysis and produce the figures in the main text.

The file **Top1000Actives_all872.csv** is an annotated listing of the top 1000 most frequently active compounds.
